# The Genus *Apis* in a Changing World: Distribution, Conservation, Climate, and Anthropogenic Stressors

**DOI:** 10.3390/insects17020185

**Published:** 2026-02-10

**Authors:** Erica Holzer, Serena Malabusini, Sara Savoldelli, Daniela Lupi

**Affiliations:** Department of Food Environmental and Nutritional Sciences, University of Milan, 20133 Milan, Italy; serena.malabusini@unimi.it (S.M.); sara.savoldelli@unimi.it (S.S.); daniela.lupi@unimi.it (D.L.)

**Keywords:** honeybees, wild bees, worldwide distribution, pollination ecology, invasion, biodiversity conservation, habitat loss, anthropogenic threats, competition

## Abstract

(1) Background: Bees of the genus *Apis* play a fundamental role in ecosystems thanks to their pollination activities and their long evolutionary history. This has resulted in species diversifying and spreading across Asia, Africa, and Europe. This review contextualises the genus within biogeographic and evolutionary frameworks, emphasising the importance of understanding the origins, adaptations, distribution and differences between species. (2) Methods: Recent studies on the biology, taxonomy and ecology of *Apis* species were analysed, including research on social behaviour, communication, genetics, morphology and environmental adaptations, as well as contributions using modern evolutionary and phylogeographic analytical methods. (3) Results: The gathered evidence shows that anthropogenic factors, including climate change, habitat loss, intensive agriculture, pollutants, competition with other bees and the spread of parasites and pathogens, significantly affect the stability of *Apis* populations and increase the vulnerability of wild species. (4) Conclusions: This review emphasises the importance of integrating ecological, genetic and management knowledge to develop effective conservation strategies that aim to reduce the impact of human activities and preserve the resilience of *Apis* species and the vital ecosystem services they provide.

## Simple Summary

Bees of the genus *Apis* represent a key taxonomic group within Apoidea, comprising species that occupy a wide range of ecological niches and exhibit distinct behavioural, physiological and colony-level traits. Their role as pollinators makes them fundamental for the reproductive success of numerous angiosperms in natural and agricultural ecosystems. In recent decades, however, *Apis* species have been increasingly exposed to different pressures. Threats due to human activities, such as habitat fragmentation, land use intensification, climate change, pollution, and the global movement of managed colonies, have altered their population dynamics and facilitated the spread of parasites and pathogens. Moreover, the mismanagement of domesticated colonies may intensify competition with wild bees, contributing to the spillover of diseases. This review synthesises the current knowledge on the distribution and origin of *Apis* species, their behaviours, and the adaptations that have enabled them to survive in diverse environments. It further analyses how human activities are disrupting this balance and the potential consequences for ecosystems and humans. Understanding these dynamics is essential for developing effective strategies to protect bees and ensure a sustainable future.

## 1. Introduction

The genus *Apis* Linnaeus (Hymenoptera: Apidae) includes some of the most studied insect species worldwide and ranks among the most ecologically, agriculturally, and culturally important pollinators [1,2,3]. These bees are valued not only for their honey, wax, propolis, royal jelly, and venom production, but also for their vital role in pollination services that underpin both wild plant reproduction and global food systems [4,5]. Species of *Apis* are known for their complex eusocial behaviour, including intricate division of labour, cooperative brood care, and sophisticated communication strategies such as the waggle dance, which allows for recruitment of floral resources over long distances [3,6,7,8,9]. Their pollination services support biodiversity in a wide range of ecosystems and are also crucial for the productivity of key agricultural crops. Several bee species have co-evolved with specific plants or play keystone roles in native floral communities [10]. Moreover, the domesticated honeybee *Apis mellifera* Linnaeus, 1758 supports annually the global economy with billions of dollars [11].

Culturally, honeybees have inspired mythologies, religious practices, and artistic expression for millennia [12,13]. In science, *A. mellifera* has emerged as a key model organism, particularly in studies of neurobiology, learning, epigenetics, and social evolution [14,15].

However, the growing ubiquity of honeybee hives, especially in urban and semi-natural environments, has raised important ecological concerns [16]. There is increasing evidence that high densities of *A. mellifera* can lead to competition with native pollinators [17], potentially altering local pollination networks and reducing floral resources for solitary and specialist bee species [18,19,20,21,22,23,24,25]. Another emerging threat lies in the horizontal transmission of pathogens and parasites such as *Nosema* spp. (currently reclassified as *Vairiomorpha)* [26], *Varroa destructor* Anderson & Trueman, 2000, and a range of viruses, between managed and wild bee populations [27]. This cross-species disease flow is now recognised as a significant contributor to global pollinator declines and presents a dual challenge for both biodiversity conservation and apicultural health [28,29].

Despite these challenges, *Apis* species continue to be extensively reared by humans as each species presents distinct traits influencing its suitability for domestication, diffusion, climate adaptability, and productivity.

This review aims to provide a comprehensive overview of the genus *Apis*, including its behaviour, ecology, and applied research perspectives. By examining both wild and managed species, their native and introduced distributions, conservation status, and interactions with biotic and abiotic stressors, this review seeks to clarify the dual role of *Apis* as both vulnerable organisms and, under certain conditions, drivers of ecological imbalance. We address how anthropogenic pressures, pathogen dynamics, and interspecific and intraspecific competition can render *Apis* populations susceptible to decline, while traits like high competitiveness, human-mediated dispersal, and disease transmission may contribute to negative impacts on native pollinator communities and ecosystem functioning.

To achieve these objectives, this narrative review was conducted through a structured survey of scientific literature to synthesise current knowledge on the ecology, biogeography, and anthropogenic impacts associated with the genus *Apis*. Literature research was performed using Google Scholar, Google, and ResearchGate to ensure broad coverage of peer-reviewed articles, reviews, and relevant manuals. Searches primarily focused on studies related to species in the genus *Apis* published within the last 25 years. Where necessary, seminal older works were included to provide historical or conceptual context. Studies were included if they addressed at least one of the following aspects: (i) diffusion, biology, behaviour, or ecology of *Apis* species; (ii) responses of *Apis* to environmental or anthropogenic stressors; (iii) interactions between *Apis* and wild pollinators, including competition and pathogen spillover; or (iv) ecological and socio-economic roles of *Apis* in native or introduced ranges. Publications focusing exclusively on technical beekeeping practices without ecological implications, anecdotal reports lacking empirical support, or studies not available in English were excluded.

## 2. Biodiversity Within the Genus *Apis*

The evolutionary history of the genus *Apis* offers valuable insights into the origin and diversification of eusocial behaviour among bees [30]. *Apis* is placed within the family Apidae and is the only extant genus in the tribe of Apini. Phylogenetic analyses, combining morphological and molecular data, suggest that the genus originated in Asia, likely during the early to mid-Miocene [31], with the most basal species still distributed across tropical and subtropical regions of southern and southeastern Asia [32].

The genus *Apis* consists of three subgenera: the dwarf honeybees (*A. Micrapis* Ashmead), the giant honeybees (*A. Megapis* Ashmead), and the cavity-nesting honeybees (*A. Apis* Linnaeus) [33,34].

Molecular phylogenies have largely confirmed the basal position of the dwarf honeybees within the genus. These open-nesting species, which construct single combs in exposed locations such as tree branches or rock ledges, are thought to represent the most ancestral traits within *Apis*. The giant honeybees, also open-nesting, form the second major clade, whereas cavity-nesting species, known for their ability to colonise enclosed spaces, form the most derived group in evolutionary terms [35]. Furthermore, the use of a standard calibration for insect mtDNA suggests that *A. mellifera* diverged from other cavity-nesters during the late Pleistocene (c800,000 years ago) [31].

The genus *Apis* is currently composed of 9 recognised species [36], which are generally classified into three major behavioural and morphological groups according to their nesting capability and to their dimension: in detail the dwarf honeybees, *Apis florea* Fabricius, 1787 and *Apis andreniformis* Smith, 1858, that are open nesting bees, the giant honeybees: *Apis dorsata* Fabricius, 1793, *Apis laboriosa* Smith, 1871 and *Apis nigrocincta* Smith, 1861, also open nesting bees, and the cavity-nesting honeybees, including the widely domesticated *A. mellifera*, as well as *Apis cerana* Fabricius, 1793, *Apis koschevnikovi* Enderlein, 1906, and *Apis nuluensis* Tingek, Koeniger & Koeniger, 1996.

Among cavity-nesting bees, *A. mellifera* and *A. cerana* are especially notable for their broad distributions and adaptation to different climates. Their divergence is estimated to have occurred around 6–7 million years ago [35,37], likely following geographic separation and climatic shifts during the Miocene. Subsequent radiation has produced numerous subspecies, especially within *A. mellifera*, whose natural range extends across Europe, Africa and parts of Western Asia [31].

According to a meta-analysis based on variations in the mitochondrial DNA sequences of 22 subspecies of *Apis mellifera*, the ancestor, *A. m. mellifera*, originated in northern Europe and diversified in south-eastern Europe before spreading to Asia Minor [31]. European bees subsequently spread southwards through the eastern Mediterranean, reaching the Nile Valley and even crossing the Red Sea. Because of the southward migration, distinct subspecies developed in Ethiopia and Madagascar. The bees found in sub-Saharan Africa belong to a single evolutionary lineage, and the separation between the Euro-African and sub-Saharan lineages dates back approximately 250,000 years. Mediterranean honeybees arrived later, around 20,000 years ago, first in the Iberian Peninsula and then in western islands and coastal areas of Africa [31].

The allopatric spread of *Apis mellifera* in the Mediterranean basin resulted in the differentiation of 33 subspecies, which are characterised by physiological, morphological and ethological traits [33,38]. These subspecies can be divided into five lineages (A (+ sub-lineage Z), M, C, O and Y) based on molecular data [38]. Specifically, Group A encompasses all the African subspecies, Group M comprises the western and northern European subspecies, Group C includes the eastern European subspecies, and Group O comprises the Turkish and Middle Eastern subspecies [36]. However, the intensive domestication of this species, particularly over the past 150 years, combined with the unrestricted commercial circulation of colonies and their intentional anthropogenic movement (e.g., nomadic beekeeping), has progressively smoothed out the differences between subspecies, even in geographical terms [38,39], through extensive hybridisation processes across nearly the entire range [40]. These dynamics have promoted uncontrolled crossbreeding among managed and feral colonies of different subspecies [41,42,43,44], resulting in a complex genetic landscape in which many subspecies are now found beyond the boundaries of their native ranges. Widespread hybridisation also represents a critical factor in genetic erosion, with a significant impact on the conservation of subspecific biodiversity [45].

### 2.1. Biogeographical Patterns and Evolution of Apis Species

**Figure 1:**
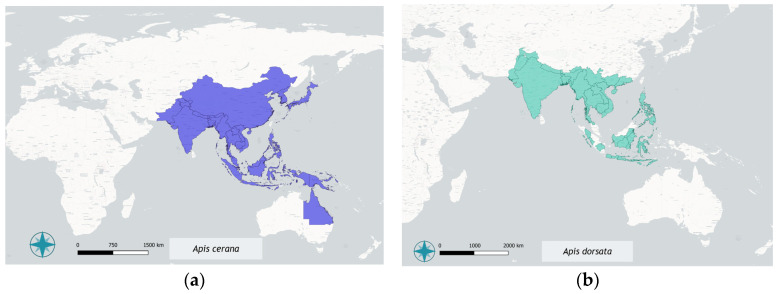

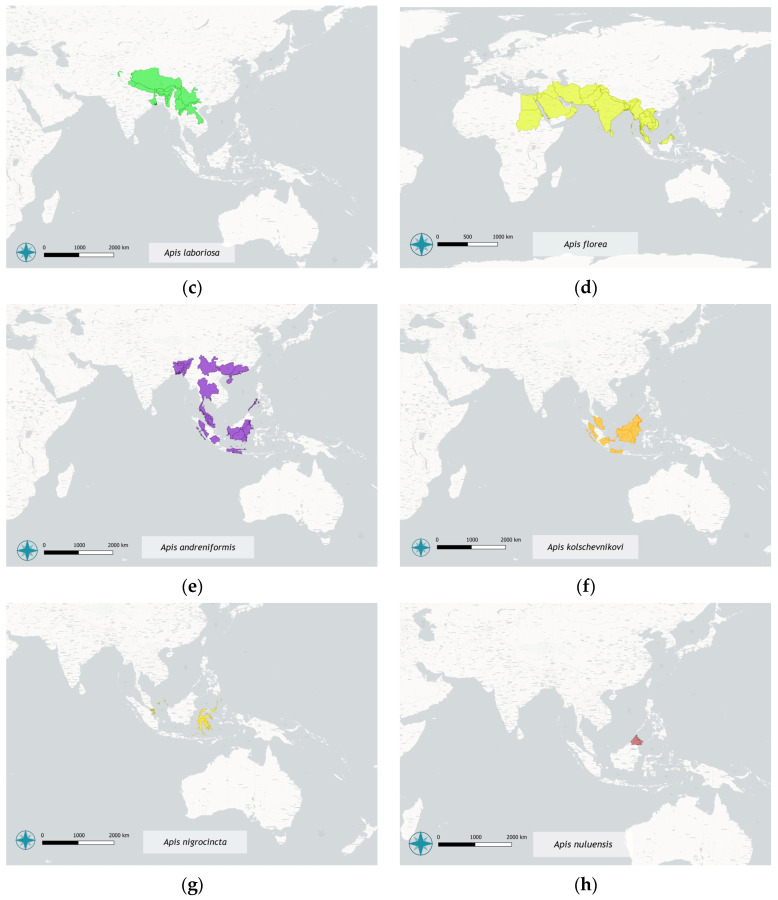
Distribution maps of (**a**) *Apis cerana*; (**b**) *A. dorsata;* (**c**) *A. laboriosa*; (**d**) *A. florea*; (**e**) *A. andreniformis*; (**f**) *A. kolschevnikovi*; (**g**) *A. nigrocincta*; and (**h**) *A. nuluensis*. Distribution data were compiled from primary literature. When the literature reported the presence of a species in a specific area within a country, the entire country was marked as positive for species occurrence. The polygons used are based on administrative boundaries from GADM v4.1 (Global Administrative Areas). The maps were created using QGIS version 3.40.5, Bratislava.

The geographical distribution of different *Apis* species has changed over time. These changes can be attributed to several factors, including the expansion of agricultural crops, climate change, and human activities [46,47]. Figure 1 shows the updated distribution maps of all the species except *Apis mellifera*, as populations of this species are present on all continents, except Antarctica [38,48]. Distribution data were derived from primary literature sources; when species presence was reported only for specific areas within a country, the entire country was conservatively considered positive, as a precise and homogeneous spatial delimitation of occurrence was not feasible across studies. A more detailed, species-specific discussion of distribution patterns and regional variation is provided for each species above in the text.

As stated, all species within the genus, except for *A. mellifera*, are native to Asia [32,35,49,50,51] and Southeast Asia and evolutionary processes have resulted in the current diverse array of honeybee species [52]. One of the earliest divergences led to the emergence of *A. dorsata* and *A. florea*, both of which constructed simple, single-comb nests that were typically exposed and offered minimal defence against predators [48]. Although their nesting strategies were relatively primitive, these two species are the closest ancestors of modern honeybees and have persisted on Earth for over 30 million years. Over time, more sophisticated nesting behaviours evolved, with specimens (the ancestors of *A. cerana* and *A. mellifera*) beginning to build cavity-based nests with multiple parallel combs. This architectural advancement, along with their capacity to cluster together and generate heat during cold seasons, allowed them to thrive in temperate climates and expand their geographic range [48,53].

The ecological niche occupied by *A. laboriosa* (Figure 1c) includes a vast mountainous area that crosses Pakistan (Azad Jammu and Kashmir), India (Uttarakhand, Sikkim, West Bengal, and the Northeast), Nepal, Bhutan, southern China (Tibet and Yunnan), Myanmar, northern Thailand, Laos, and Vietnam [54,55]. The species is adapted to the cold, mountainous environments of South Asia, able to survive at altitudes ranging from 1000 to 3600 m, although nests have also been documented at lower altitudes (down to 800 m) in north-eastern India [56]. It prefers exposed rock faces for nesting, and it is a key pollinator of subalpine ecosystems as it can visit many endemic plants, being active even at low temperatures and in harsh environmental conditions [57,58]. *Apis laboriosa* nests in highly inaccessible environments, such as steep rock faces in the Nepalese mountain range [59]. These characteristics render the species unsuitable for domestication and conventional beekeeping practices, but colonies are traditionally exploited for honey gathering [60].

The distribution range of *A. dorsata* (Figure 1b) extends from Pakistan and India through the Himalayas to the Philippines in the east. It passes through most of Indonesia and East Timor and extends north to western and southern China [56]. This migratory and highly adaptable species nests in large, exposed colonies, often on tree branches or artificial structures. It is renowned for its impressive single-nest constructions. It is ecologically important for both honey production and the pollination of numerous plant species in tropical habitats; in addition, it is considered one of the most efficient bees for foraging in low- and medium-altitude environments [61,62].

*Apis florea* (Figure 1d) is native to South and Southeast Asia, where it is widely distributed across tropical and subtropical environments. Its natural range extends from Vietnam in the east to Iran, Iraq and Oman in the west, and encompasses countries such as India, Sri Lanka and Thailand [56]. While it prefers subtropical climates, it can colonise a variety of habitats, including tropical rainforests, savannas, subtropical grasslands, and semi-deserts. In Southeast Asia, *A. florea* coexists with another species of dwarf bee, *A. andreniformis.* However, *A. florea* shows greater ecological flexibility and has expanded its geographical range more markedly. Traditionally confined to continental Asia, its range has recently expanded southwards to reach the Malay Peninsula and Singapore, and westwards to establish itself in areas such as Saudi Arabia, Israel, Jordan, North Africa and Egypt, where it is considered an alien species [63,64]. This expansion has largely been attributed to anthropogenic factors, particularly international trade and maritime transport [56]. It is predicted and feared that it may further expand its range in the future, potentially establishing itself in parts of tropical Africa, probably due to several factors, including unintentional transport by aircraft or cargo ships, and ongoing climate change [64,65,66,67,68,69]. The stable establishment in Jordan, combined with the absence of significant geographical barriers, raised concerns about the possible further spread to Asia Minor and potentially Europe. This scenario materialised in May 2024 with the first documented case of stable colonisation in Europe, when a colony of *A. florea* was observed in Malta [68]. This was most likely the result of a post-division swarm. In 2022, a swarm from India was intercepted and removed at Genoa port (Italy) [68].

*Apis andreniformis* (Figure 1e), also known as the black dwarf bee, is a strictly tropical species adapted to living at low altitudes. It is found in some areas of Southeast Asia, where its range partially overlaps with that of *A. florea*. Its range extends from Bangladesh and north-east India to southern China, the Malay Peninsula and the islands of the Sunda Shelf, including Sumatra, Java, Borneo and Palawan [56]. Unlike *A. florea*, *Apis andreniformis* is absent in cold or arid environments. It is also not present in the wild on islands that have never been connected to the mainland, such as those in the Philippines, the Andaman Islands, Sulawesi and the islands from Lombok to Timor. This is probably due to its limited ability to disperse across deep marine barriers [63]. This indicates a low propensity for natural geographical expansion, in stark contrast to its congener. Colonies of *A. andreniformis* are typically found at altitudes below 1000 m, and their single honeycomb nests are built in sheltered spots within vegetation. Ecologically, the species plays a crucial role as a pollinator in many parts of Southeast Asia [70] and is considered a fundamental component of the agricultural and forestry ecosystems of tropical regions due to the services it provides [71].

*Apis cerana* (Figure 1a) is widely distributed across South, Southeast and East Asia. Its natural range extends from Pakistan and Japan to the Philippines, Indonesia and the Siberian region of Primorye [33,63,72]. It has adapted well to a variety of habitats in both temperate and tropical environments, ranging from northern Afghanistan to the foothills of the Himalayas and Southeast Asia [73]. It is also present in Papua New Guinea. This species also spread accidentally, reaching the Solomon Islands and Australia, where it established in Queensland, in the north-eastern Country [72,73]. Although attempts have been made to stop the spread of this species, its distribution area is still expanding [74,75].

The mitogenomic phylogeography of *A. mellifera* was reconstructed by Carr [31], revealing a predominantly north-to-south expansion from Europe to Africa via Asia Minor and the Levant. The nominal subspecies *A. m. mellifera* likely originated in northern Europe, from which *A. m. ligustica* diverged in southeastern Europe. The latter expanded eastward into Asia Minor, giving rise to *A. m. caucasia*. Continued southward dispersal through the Levant produced *A. m. syriaca*, followed by expansion into the Nile Valley and the emergence of *A. m. lamarckii*. The lineage then crossed the Red Sea to the Arabian Peninsula, leading to *A. m. jemenitica*, and subsequently diversified further south into early-branching African subspecies such as *A. m. simensis* in Ethiopia and *A. m. unicolor* in Madagascar. Sub-Saharan taxa (including *A. m. scutellata*, *A. m. capensis*, *A. m. adansonii*, and *A. m. monticola*) form a single clade, with *A. m. adansonii* widespread in central Africa (though genomic representation from West Africa remains limited). The Mediterranean clade appears to reflect a secondary northward return to Europe during a glacial refugium period, followed by a later dispersal into North Africa through western Mediterranean islands. Overall, Africa contains the greatest diversity of *A. mellifera* subspecies, highlighting its central role in the evolutionary history of the species. Today, it occurs on every continent except Antarctica, inhabiting diverse environments from tropical to temperate regions. Managed and feral colonies are widespread in the Americas, Australia, New Zealand, and large parts of Asia [48].

*Apis nuluensis* (Figure 1h) is endemic to the highlands of Mount Kinabalu in the Malaysian state of Sabah on the island of Borneo. It predominantly occurs above 1700 m, where it is the only *Apis* species reported in systematic surveys between 1700 and 3400 m [56,76]. In the intermediate zone (1500–1700 m), it coexists with *A. cerana* and *A. koschevnikovi,* exploiting the same floral resources without evidence of hybridisation [76]. It was first described as a new species by [76], and morphometric analyses conducted by [77] highlighted the characteristics that allow it to distinguish A. *nululensis* from other sympatric species, confirming it as a distinct species. However, later on it was suggested to be considered a subspecies of *A. cerana*, emphasising the need for further research [32]. Subsequent molecular analyses have confirmed again this genetic distinction by reveal; ing unique haplotypes and a high degree of divergence from other Asian *Apis* species [45,78]. In the end, more recently, the complete mitochondrial genome of *A. nuluensis* was analysed for the first time to clarify its phylogenetic position and genetic distance from other species of the Asian *Apis* complex [79].

*Apis koschevnikovi* (Figure 1f) is distributed in the Sundaland region, which includes the Malay Peninsula and the islands of Sumatra, Bangka, Java, and Borneo [56]. In peninsular Malaysia, it is bounded to the north by the Kangar-Pattani line [80]. Originally described by Enderlein in 1906 (as *Apis indica* var. *koschevnikovi*), it remained poorly documented for almost eighty years, until its rediscovery in Borneo in the 1980s [81]. Subsequent morphometric and genetic studies confirmed the specific identity of *A. koschevnikovi*, which is distinct in terms of morphological characteristics [81], reproductive isolation [82], and nuclear and mitochondrial DNA sequences [52,78]. Analysis of its morphometric variation on a geographical scale has made it possible to define its population structure and trace its biogeographical relationships with other sympatric species: *A. cerana* and *A. nuluensis* [83].

*Apis nigrocincta* (Figure 1g) is a species with a restricted distribution, found mainly on the highlands around mount Kinabalu (Borneo), at altitudes above 1500 m. In addition, it has been reported on the Indonesian islands of Sulawesi, Sangihe, and, with less certainty, on other nearby islands [56,80]. Its limited distribution and insularity make it one of the most ecologically specialised species in the *Apis* genus.

### 2.2. Domestication Process of Honeybees

Domestication has primarily involved cavity nesting bees and in detail *A. mellifera* and *A. cerana*, whose successful spread can be attributed to their ability to adapt to a wide range of ecosystems and climates, as well as to direct human intervention. In fact, in order to exploit the hive products resulting from the work of these bees, humans have introduced them to areas where they did not occur naturally, selecting individuals that adapted best to the new conditions and effectively turning into alien, and sometimes invasive, insects [67,73,84,85,86,87,88].

The earliest evidence of human interaction with bees relates to honey hunting rather than beekeeping. Iberian rock paintings from 7000 to 8000 years ago show people collecting combs from wild nests, and chemical traces of bee wax in Anatolian pottery nearly 9000 years old further demonstrate early use of bee products [89]. Honeybee domestication represents one of the earliest forms of insect management and has played a central role in agricultural development [48]. Domestication was possible in areas where cavity-nesting honeybees were settled. The earliest evidence of honeybee domestication dates to ancient Egypt, when bees were reared in colonies as depicted in some hieroglyph [12]. Following this, traces of honeybees’ management were found in all the great societies of the past (e.g., ancient Greeks or the Romans) [90].

Due to the significant value of honeybee products, several species of *Apis* have expanded their range through human-mediated introductions. Consequently, current distribution patterns are the result of both natural evolutionary processes and human intervention. Although the Americas were once believed to lack native *Apis* species, fossil finds in Nevada suggest early honeybees may have crossed from Asia the land bridge thought to connect modern-day Russia to Alaska, but these early populations likely went extinct long before human arrival [91].

*Apis mellifera* was then introduced in America by European settlers in the 17th century and, since then, has widespread throughout both continents [46,92]. *Apis mellifera* has also been introduced to Australia between 1810 and 1820–1822 for honey production and pollinating agricultural crops [93,94,95,96].

In 1956, *Apis mellifera scutellata* was introduced into Brazil with the aim to create hybrid well suited to South America’s tropical and subtropical climates that retained the docile and productive characteristics typical of *A. mellifera ligustica*. However, the resulting hybrids, known as Africanised honeybees, escaped control and spread rapidly across the American continent reaching Central America in 1982 and United States in 1990 [97,98,99]. Recent reports of swarms of Africanised bees in Alabama, on the border with Georgia, are evidence of this spread [100].

*Apis cerana*, the Eastern honeybee, is the earliest honeybee species managed by humans in Asia. Traditional beekeeping has involved log and box hives, allowing the species to nest in conditions like natural cavities [63]. Over centuries, human management favoured colonies that tolerated disturbance and provided honey and wax, although domestication has remained largely traditional and low-intensity compared to *A. mellifera* [51]. The species was reared for up to two thousand years in Asia, just recently (XX century) it was replaced by the most productive *A. mellifera* [48,101]. Despite the reduced productivity *A. cerana* was also intentionally introduced into areas outside of its natural range. Specifically, it was introduced to Papua New Guinea in the 1970s. It is widely distributed across South, Southeast and East Asia [101]. *A. cerana* provides also essential pollination services for Asian agricultural and natural ecosystems and is tightly linked to local floral resources and cultural practices [102,103,104,105]. Moreover, its long co-evolution with regional pathogens and parasites, including *Varroa* spp., has shaped its behavioural defences and colony dynamics, offering valuable insights into honeybee resilience and locally adapted apiculture strategies [73,106].

The cavity-nesting honeybee group includes also other species of *Apis*, which are distributed throughout southeast Asia (*Apis negrocincta*, *Apis nuluensis* and *Apis koschevnikovi*), endemic and geographically restricted, that deserve special attention due to their ecological importance and conservation implications but less from a productive point of view [76,80,81,107,108].

From an economic point of view, also the gathering of *A. laboriosa*’s honey plays an important role in high-altitude honey production, especially in Nepal, where its annual productivity can vary between 20 and 100 kg per colony [109]. This honey is highly prized locally and has also significant cultural and commercial value [110,111].

## 3. Anthropogenic Pressures on Apiculture

Intensive beekeeping practices and human interference with natural environments generate a complex set of pressures that compromise both honeybee management and the survival of wild bees and other pollinating insects [22]. These pressures can be broadly divided into two categories [112]. The first includes direct anthropogenic factors, such as habitat fragmentation, chemicals contamination, and climate change, which affects ecosystems and reduces the availability of suitable foraging and nesting sites [113,114]. The second encompasses the introduction, spread, and spillover of parasites, pathogens, and alien species which further exacerbate pollinator decline and colony health issues [115,116,117]. These negative stimuli act synergistically, compromising both managed colonies and indigenous populations.

### 3.1. Habitat Fragmentation and Loss

Converting natural environments into intensive agricultural, urban or industrial areas leads to a loss of continuity and quality in the habitats where bees can nest and forage. This fragmentation reduces the number and size of areas of semi-natural vegetation, thereby it increases the distance between foraging and resting/nesting areas and consequently raises the energy costs for pollinators (both solitary and social). In agricultural landscapes, for instance, reducing forest cover or semi-natural vegetation decreases the diversity and abundance of wild bee species [118,119,120,121,122]. Bees of the genus *Apis* depend heavily on the availability of spatially heterogeneous resources; fragmentation increases their foraging energy costs and reduces their ability to maintain a positive balance between resource collection and consumption [122,123,124,125]. Open-nesting bees, such as *A. dorsata*, nest in exposed environments or in the cavities of large trees. They are therefore particularly vulnerable to the removal of trees and the loss of mature specimens, which results in a decrease in nesting opportunities [61]. Furthermore, habitat fragmentation can lead to competition for resources between farmed and wild species. For example, the introduction of *A. mellifera* into non-native ecosystems, can reduce the fitness and productivity of local species of *Apis* and other Apoidea [126,127,128]. Habitat loss intersects with other pressures: fragmented areas are more susceptible to the effects of climate change, such as reduced plant diversity and fewer protective microclimates, as well as increased contact with chemicals and pathogens. Therefore, habitat fragmentation should be considered as one component of a broader context of environmental stresses.

### 3.2. Pollution and Agrochemicals

Exposure to environmental pollutants and the intensive use of agrochemicals represent additional stress factors for *Apis* populations. Different insecticides and systemic fungicides, can interfere with the central nervous system of bees, thereby compromising their olfactory memory, orientation, and learning ability [129]. As an example, neonicotinoids act agonistically to nicotinic acetylcholine receptors (nAChRs), which interferes with normal synaptic transmission in the bees’ central nervous system and causes neurotoxic effects that compromise flight behaviour, orientation and foraging efficiency [130,131,132]. Even sublethal exposure to these compounds significantly reduces foraging and spatial learning abilities, resulting in disorientation and difficulty returning to the hive. It also increases forager mortality due to loss of motor coordination and physiological deterioration caused by oxidative stress and neuronal dysfunction [133]. In addition to agrochemicals, air and heavy metal pollution can alter the chemical signals that bees use for intra-colony communication. This reduces recruitment effectiveness and increases disorganisation within the hive [134,135]. The presence of microplastics and volatile organic residues has recently been linked to sub-lethal effects on the respiratory physiology and gut microbiota composition of bees. These effects can have long-term consequences for the bee immune system [136]. In intensive agricultural settings, the effects of chemicals are exacerbated by the scarcity of floral resources and monoculture, reducing pollen diversity and increasing colonies’ dependence on contaminated sources [137,138,139,140]. These cumulative effects result in a progressive decline in colony survival and reproductive success, particularly in wild and unmanaged bee populations. Several experimental studies demonstrate that ‘multi-stress’ conditions result in higher mortality rates and physiological and behavioural alterations in both farmed and wild bees [141,142]. Recent studies have shown that exposure to both agrochemicals and electromagnetic fields simultaneously can cause physiological and behavioural changes in bees. This interferes with their orientation processes and foraging efficiency, accentuating the cumulative effects of other environmental stresses [142]. These conditions result in a gradual decrease in the survival of bee colonies and a reduction in their reproductive success, particularly in wild and unmanaged populations, which are more susceptible to ecological disturbances and limited food resources [138,143,144,145]. Exposure of *A. mellifera* brood, adults and hive products to insecticide and miticide residues has been documented in different studies [139,146,147]. In response to regulatory requirements, the European Food Safety Authority (EFSA) updated its guidelines for assessing the risk of plant protection products to bees (including wild bees) in 2024. The updated guidelines aim to implement legislation and the evaluation process for plant protection products and biocides, considering knowledge that has emerged over the last decade [148].

*Apis cerana* is also highly vulnerable to insecticides and other environmental contaminants, particularly in intensive agricultural settings where regulations are less stringent than in Europe. A recent study in the context of combined exposure showed that administering imidacloprid, chlorpyrifos and glyphosate together significantly reduced the flight capacity of *A. cerana* [149]. Furthermore, ref. [150] documented the presence of insecticide residues in bee products, including bee bread and honey.

The literature addressing the effect of chemicals on *A. dorsata* and *A. laboriosa* is limited, but both species inhabit forests and mountains that are becoming increasingly fragmented due to the conversion of large areas for agricultural use. A review paper of [108] emphasised that *A. laboriosa* is under threat from changes in land use and destructive harvesting of colonies. Sihag [61] draws attention to the sharp decline in *A. dorsata* populations, which is attributed to the loss of nesting sites and mining practices. This suggests that the introduction of agrochemicals and pollution may act as an aggravating factor.

### 3.3. Effects of Climate Change

Climate change is an additional destabilising factor for the genus *Apis*, altering the phenology of plants, the seasonal availability of resources, and the microclimatic conditions of nesting habitats [151,152]. Rising global average temperatures and irregular rainfall directly impact foraging cycles, reproductive activity and the survival of colonies [153,154]. Furthermore, extreme weather events such as heatwaves and prolonged droughts reduce the availability of nectar and pollen [113,155]. This limits the ability of colonies to accumulate sufficient resources for maintenance during critical periods [156,157].

The effects of global warming manifest differently among species [158,159]. *Apis mellifera* exhibits behavioural and physiological plasticity, whereas open-nesting bees, which are more closely linked to specific temperature and humidity regimes, are less adaptable [51,153]. Climate-based distribution models predict for *Apis dorsata* a severe contraction of this range in future climate scenarios in northern high-altitude areas [160]. On the contrary, *Apis florea* may benefit from rising temperatures and milder winters, which facilitate range expansion into areas that were previously climatically unsuitable [64,65,66,67,68,69].

Climate change also indirectly impacts the health of *Apis* populations by promoting the proliferation of pathogens and parasites, as well as the geographical expansion of invasive species, with dramatic consequences [153,161,162].

For the most managed and studied species, *A. mellifera*, changes in seasonal temperatures, an increased frequency of extreme weather events and changes in the phenology of honey plants pose direct and indirect risks [152,153]. Some studies have shown that rising temperatures prolong the bee activity phase and favour infestation by *V. destructor*, as colonies remain active for longer in conditions that are favourable to the mite [153,163,164]. This results in poorer winter survival [156,163]. Recent studies further indicate that warmer winters and extended brood-rearing periods disrupt the natural break in the reproductive cycle of *V. destructor*, allowing mite populations to increase continuously throughout the year [164,165]. In addition, altered flowering phenology and increased climatic unpredictability can lead to nutritional stress, weakening colony immune responses and increasing susceptibility to parasites and pathogens [113,157]. Together, these factors create synergistic stressors that amplify colony losses and compromise the long-term resilience of *A. mellifera* populations under ongoing climate change [153,165,166].

## 4. Competition Between Honeybees and Other Hymenoptera Pollinators

Competition between honeybees and other pollinators is an ecologically significant phenomenon, particularly in regions where species bred for producing bee products have been introduced beyond their native habitat [18,19,167,168]. *Apis mellifera* and *A. cerana* are naturalised in multiple regions worldwide and have sometimes displayed invasive behaviour [67,73,84,85,86,87,88].

A recent systematic review of 96 studies found evidence of exploitative competition by *A. mellifera* towards wild pollinators in 78% of cases [169]. However, only 13% of the studies assessed the effects on the fitness of individual wild pollinators, and, surprisingly, more than 60% found no negative correlations [170]. In contrast, another study [171] revealed a negative correlation between the abundance of *A. mellifera* and the diversity of wild bee species in urban areas, particularly among smaller species with intertegular sizes under 2.25 mm. This suggests that small wild bees, with limited foraging ranges, are more vulnerable in areas with a significant honeybee population. A study of the functional traits of plants and pollinators [172] revealed that the likelihood of competition between *A. mellifera* and wild pollinators is influenced by the similarity of their foraging habits and access to the same floral resources. Basically, if *A. mellifera* and local pollinators choose similar plants and flowers, there is more chance of them competing. This thesis is also supported by other research studies [173,174]. Using palynology [175] and DNA metabarcoding, the authors investigated whether managed honeybees and wild bees compete for pollen in three Italian National Parks. The results indicated high resource partitioning, suggesting low trophic overlap and limited direct competition in these biodiverse habitats. This supports the idea that competition depends on context [175].

The introduction of *Apis* species into non-native regions, as well as the increase in honeybee hives in human areas, has also raised ecological concerns, especially regarding competition with native pollinator communities, as well as changes in local pollination networks [20,25,126]. In the case of the introduced range of *A. mellifera* in South America, the species may play an even more critical role: as it has been introduced, it cannot co-evolve with the local flora in the same way as native pollinators. For example, a study on the pollination mechanisms of the dioecious tropical tree *Clusia mexiae* P.F.Stevens (2017) (formerly *Clusia arrudae* Planchon and Triana, 1860), which is widespread in the ‘campos rupestres’ of the Brazilian Coastal Atlas, found that male flowers produce large amounts of pollen (approximately 11 million grains per flower over three days of flowering) and that female plants remain receptive for several days if pollination does not occur within the first few days [176]. The researchers observed that visitors to the male flowers included *A. mellifera*, as well as several native species that have co-evolved and specialised in pollinating this plant. However, *Apis mellifera* was found to be responsible for removing 99% of the pollen. This resulted in a significant reduction in the pollination success of *C. arrudae*, as honeybees collected large quantities of pollen but contributed relatively little to effective pollination. Other scientific evidence includes a study by [177], which highlights the negative effects that the high density of managed colonies of *A. mellifera* can have on the diversity and abundance of native bees. The study monitored 11,520 pan traps over three years and revealed the predominance of honeybees at the expense of other species. Other studies, conducted in Brazil and other Latin American countries by [178], have shown a significant negative impact on native bees of the Africanised bee through competition for floral resources. A review about the introduction of *A. cerana* in Australia mentioned the concerns about the impact on indigenous fauna and flora, as well as on managed *A. mellifera* colonies (e.g., hive robbing) [73]. *Apis cerana* is potentially a competitor of native pollinators in introduced areas, but few studies evaluate its impact, and in native areas, competition appears to be mitigated by co-adaptation and specialisation. To support this theory, *A. cerana* was reported to be able to forage from numerous sources in a wide temperature range (5–45 °C), giving it a competitive advantage in modified environments [73,179]. On the contrary, in contexts where *A. cerana* is native, such as Japan, the species appears to be relatively resilient to land use change, suggesting reduced competition in conditions of historical coevolution [180].

After the recent settlement of *A. florea* in the Nile area in Sudan, the species has reached densities that exceed those of the local *A. mellifera,* although without direct evidence of competitive displacement [68,181]. However, other studies have emphasised the invasive nature of *A. florea*, due to its ability to adapt to arid climatic conditions and its tendency to swiftly colonise altered or marginal ecological niches [181,182].

## 5. Transmission of Pathogens, Viral Diseases, and Spillover

Different problems affect honeybee survival, including bacterial and viral diseases, microsporidian infections, pests and predators. The extensive documentation of pathogens in honeybees, including over seventy different species of viruses [183], bacteria [184], and microsporidia [185], reveals the complexity of the threats these insects face. A recent review [186] updated the knowledge on the routes of virus transmission in honeybees, showing that both horizontal and vertical pathways are involved. Among the vectors, the mite *V. destructor*, originally associated with *A. cerana*, is the primary driver of viral epidemics in honeybee populations for different viruses. The mite worldwide invasion process and its close relationship with honeybees have intensified viral infections, contributing to their spread through colonies and increasing the viral load [187,188]. In detail, the most important viruses are the Deformed Wing Virus and Acute Bee Paralysis Virus [183,189]. In addition, *Nosema (Vairimorpha) ceranae* Fries et al., 1996, a microsporidian native to Asia and originally associated with the Asiatic honeybee *A. cerana*, has emerged as an infectious disease affecting *A. mellifera* globally [190]. The small hive beetle, *Aethina tumida* Murray, 1867 (Coleoptera: Nitidulidae), is another invasive pest of *A. mellifera* colonies that feeds on brood, pollen, and honey, causing fermentation of stored products and colony weakening or collapse [191]. Also, predation by Vespidae represents a serious and growing threat to honeybee colonies. In particular, *Vespa velutina* Lepeletier, 1836; native to Southeast Asia, where it cohabits with *A. cerana*, is now invasive in several countries across Europe. Following its introduction in France in the early 2000s, has rapidly expanded its range across Western and Southern Europe, with continuing spread towards northern and eastern regions [192,193,194,195]; preying intensively on *A. mellifera* workers, often stationing near hive entrances and capturing returning workers or entering into the hives. This predatory pressure disrupts foraging activity, reduces food intake, and can ultimately lead to colony weakening or collapse, especially in areas where *V. velutina* populations are expanding [196].

A growing concern is about the horizontal transmission of pathogens and parasites, and various viruses among managed and wild bee populations [29]. This cross-species disease flow is now recognised as a major component of pollinator decline and a challenge for both conservation and apicultural health [1,97]. Many of the pathogens and parasites infecting honeybees are also capable of infecting other pollinator species [28,29,117,197,198], significantly contributing to the observed decline in wild bee populations [126,199]. Pathogen spillover is facilitated in ecological contexts where managed and wild pollinators co-occur and share resources, such as Flowering crops, semi-natural habitats, cities and areas surrounding apiaries [198,200,201,202]. Infected bees frequently visiting flowers lead to flower surfaces, nectar, and pollen becoming contaminated with virus particles during foraging, providing opportunities for transmitting multiple pathogens. Even non-susceptible visitors may act as mechanical vectors, harbouring and spreading pathogens during routine foraging [203]. Finally, horizontal transmission can be mediated by vectors such as parasitoids, parasites, and commensals that live in, on, or with the host bee or its nest structures, providing another route for pathogens to move across species [198]. As a consequence, pathogen flow is typically bidirectional: while managed honeybee colonies often act as sources, transmitting viruses to wild pollinators, wild bee populations can also act as reservoirs, contributing to pathogen circulation back into managed colonies. Additionally, habitat simplification and fragmentation can further concentrate pollinator activity, increasing interspecific contact rates and exposure risk [29]. However, for numerous solitary wild bee species, the role of these infectious agents in their survival remains poorly understood. For example, while it is known that wild bumblebees can be adversely affected by Deformed Wing Virus (DWV), Kashmir Bee Virus (KBV), and Israeli Acute Paralysis Virus (IAPV) [197,204,205], the impact of such viruses in other solitary bees is largely unexplored [206,207]. Recent advances in genomic and virome-based approaches have provided critical insights into the mechanisms underlying pathogen spillover among pollinators. High-throughput sequencing and comparative phylogenetic analyses have revealed genetic differentiation of RNA viruses [208], shared viral haplotypes and low across managed and wild bee hosts, indicating frequent cross-species transmission rather than long-term host specialisation [198,209,210,211]. Metavirome studies further demonstrate that pollinator communities often harbour complex, overlapping viral assemblages, with infection patterns strongly associated with ecological contact rates, host density, and resource sharing, rather than host taxonomy alone [212,213].

*Nosema ceranae* has also been documented to spill over into wild bee species such as different stingless bee species [210,214] and bumblebees [211]. Direct effects have been recorded, such as reduced lifespan in the Australian stingless bee *Tetragonula hockingsi* (Cockerell, 1929) [210], and minimal apparent harm to the Eurasian bumblebee *Bombus terrestris* (Linnaeus, 1758) [215] and the European mason bee *Osmia bicornis* (Linnaeus, 1758) [216]. Also, the pest *A. tumida* can move from honeybees to bumblebees colonies [191].

## 6. Discussion and Conclusions

This review article aims to provide an updated and comprehensive overview of the current knowledge on species diversity within the genus *Apis*, as well as the issues that compromise and complicate the management of domesticated bees, and contribute to the decline of bees, including wild species belonging to the same genus.

The information primarily derives from *A. mellifera*, for which the available information is most abundant and robust, allowing it to be used as a reference model to interpret stress responses in other *Apis* species. Nevertheless, a substantial body of information was also retrieved for non-*mellifera* species, highlighting both shared and species-specific patterns of vulnerability and resilience to environmental stressors.

Regarding the current distribution of the different *Apis* species, recent articles have been examined to document the ways and speed with which some species are expanding due to anthropogenic and environmental factors.

Similarly, the sections dedicated to factors that are affecting bees are based on knowledge established over the last few decades, with the state of knowledge being updated using data from recent studies. Overall, evidence spanning habitat fragmentation, chemical and electrochemical pollution, and climate change indicates that these stressors act synergistically rather than independently, jointly undermining the resilience of *Apis* populations. The strongest and most consistent empirical support concerns *A. mellifera*, for which laboratory, semi-field, and long-term field studies converge in demonstrating impaired foraging, cognition, immunity, and colony survival, particularly under combined exposure to agrochemicals, nutritional stress, parasites and diseases. By contrast, evidence for non-*mellifera* species, especially open-nesting bees but also for *A. cerana*, is more limited and often indirect, with population declines primarily inferred from habitat loss, land-use change, and destructive harvesting rather than controlled stressor experiments. Findings on climate change resilience are partly contradictory: while *A. mellifera* shows some behavioural and physiological plasticity, increasing climatic variability and warmer winters consistently intensify parasite pressure and nutritional stress. Major knowledge gaps remain regarding sublethal chronic multi-stressor effects under realistic field conditions, species-specific sensitivity to emerging pollutants, and the extent to which competition with managed honeybees exacerbates stress in fragmented landscapes. Addressing these uncertainties is essential for improving risk assessment frameworks and developing conservation strategies that reflect real-world ecological complexity.

## Data Availability

No new data were created or analysed in this study.
